# Can engineers represent surgeons in usability studies? Comparison of results from evaluating augmented reality guidance for laparoscopic surgery

**DOI:** 10.1016/j.cag.2024.01.008

**Published:** 2024-04

**Authors:** Soojeong Yoo, João Ramalhinho, Thomas Dowrick, Murali Somasundaram, Kurinchi Gurusamy, Brian Davidson, Matthew J. Clarkson, Ann Blandford

**Affiliations:** a Wellcome ESPRC Centre for Interventional and Surgical Sciences, University College London, London, United Kingdom; b UCL Interaction Centre, University College London, London, United Kingdom; c Division of Surgery and Interventional Science, University College London, London, United Kingdom

**Keywords:** Laparoscopic surgery, Augmented reality, Operating theatre, Usability study

## Abstract

Obtaining feedback from time-constrained end-users is a major challenge in evaluating novel systems for specialised applications. The performance and feedback of engineers and surgeons was evaluated through an experiment where participants were asked to identify tumour locations within an anatomically realistic silicon liver model across three different conditions of an Augmented Reality (AR) prototype system (Baseline, Split AR and Full AR). Our findings show that engineers and surgeons share some similarities in their performance, feedback and behaviour, particularly when reliance on the AR system is high for both groups. However, engineers typically focus more on accuracy of the image alignment and are more accurate in their responses when supported by AR. Senior surgeons typically perform faster and use AR as supplementary information, while the performance of junior surgeons is more closely aligned to the performance of engineers. We conclude that engineers could be involved in preliminary evaluations of a surgical system or in evaluations of systems which are aimed at training junior surgeons, but that it is essential to involve surgeons in later evaluations, where ecological validity is a more important consideration.

## Highlights

• Level of reliance on a system and expectations around system accuracy can differ between engineers and surgeons. Where surgeons (particularly senior surgeons) were faster and use augmented reality (AR) as supplementary information and engineers were more accurate when using AR system.
• We found that testing with engineers could be an ideal alternative when testing digital systems during preliminary stages.
• This work recommends that engineers should not replace testing technologies with experienced surgeons, rather this method should only be used as an early indicator in the preliminary stages of a research project.
• Engineers could also be involved in evaluations of systems which are aimed at training junior surgeons, but it is essential to involve surgeons in later evaluations, where ecological validity is a more important.

## 1 Introduction

Laparoscopic surgery, also known as keyhole surgery, allows surgeons to access the abdominal cavity without making a large incision in the skin. For the particular case of liver tumour resection, this approach has known benefits in terms of reduced trauma and recovery time for the patient and reduced costs to the healthcare system [1]. However, the range of movement of laparoscopic tools inside the body is limited, the surgeons do not have haptic feedback (e.g. cannot palpate the liver to find lesions), and the visibility of the surgical scene is restricted to a 2D display captured by the laparoscopic camera (laparoscope). Therefore, only 5%–30% of patients are considered for laparoscopic surgery, mainly when tumours are in easily accessible locations [2].

Augmented reality (AR) has been proposed as an image guidance technique to increase the safety of the procedure [3]. AR is a technology that enables virtual information to be overlaid or integrated within real world environments, commonly experienced through head mounted displays (HMD) or smartphones/tablets. By overlaying a 3D model of the liver onto the laparoscopic camera view, surgeons can have “x-ray vision” to view the relevant internal anatomy such as blood vessels and tumours. It provides laparoscopic surgeons with more context on the position of critical structures which should not be damaged, leading towards more informed decisions in the operating theatre [4]. Compared to HMD [5] such as the Microsoft HoloLens,^1^ AR in laparoscopic surgery has different hardware requirements as instead of directly seeing the target organs, surgeons can only view the surgical scene via the laparoscopic camera monitor. For that reason, a tracking system [6] needs to be used to reliably establish accurate positioning between the virtual 3D representation and the laparoscopic camera view. These additional hardware requirements complicate the clinical implementation of this technology.

A key challenge for Human–Computer Interaction (HCI) research which explores the usage and design of these technologies is the early stage development and testing with surgeon participants, who are difficult to recruit due to time constraints from their demanding schedules [7], [8], [9]. Furthermore, given the logistical challenges in coordinating surgical teams and operating theatre time slots, very few studies on surgical AR have been conducted with surgeons in an ecologically valid environment [10] (the theatres where they actually operate). Instead, a mock operating theatre environment is usually considered, and the recruited participants do not always have a medical background or any surgical experience [11].

Therefore in cases where surgeons are difficult to recruit and a surgical system is in the early stages of development and needs to be rapidly evaluated, it would be helpful to better understand **whether non-surgeons can be participants in place of surgeons**. In HCI research, it is often easier to recruit engineers—specifically, those individuals who design and develop prototypes for medical devices in research—because of their relative availability and non-clinical familiarity with relevant devices and tasks. We conducted a study with 24 participants, 12 surgeons with varied levels of experience in laparoscopy and 12 engineers, and evaluated the usability of an AR guidance system prototype called Smartliver [12] for locating tumours in a simulated surgical procedure. We analysed participant results across Usability, User Feedback, and Tumour Localisation Performance. Based on this we discuss the key differences between the two groups.

As the first study to compare the performance and qualitative feedback of engineers and surgeons in a simulated medical environment, our contribution identifies the differences between these two groups when recruited for usability studies to understand when and how engineers can represent surgeons.

## 2 Related work

### 2.1 Evaluating novel AR systems for surgery

Research has long been exploring the potential of applying novel technologies such as AR for use by surgeons [3], [13], [14], [15], [16]. AR has brought benefits to surgeons, such as augmenting their view within operating theatres and improving their efficiency [17], [18]. For the particular case of laparoscopic liver surgery, many authors have sought to understand how AR can be used to make a surgeon’s job easier and safer by overlaying information on the laparoscopic video feed, such as a preoperative 3D model obtained from an individual patient’s CT scan [19], [20] or live ultrasound images of the liver [21]. However, usability studies on novel technologies for laparoscopic surgery are limited, and focus on simpler laparoscopic display aspects rather than AR. For instance, Kumcu et al. [22] studied the effect of video latency on surgical performance with surgical trainees and surgeons, using a laparoscopy home-trainer; Van Veelen et al. [23] evaluated the effect of the laparoscopic display position in a study with a surgeon cohort; Walczak et al. [24] performed a similar analysis, but with medical students and a laparoscopic simulator; and Lim et al. [25] tested the use of glasses for display versus a conventional screen, in real surgical cases. The closest study that pertains to the usability of AR in liver surgery was presented by Schneider et al. [12], where different techniques to align the 3D surface of the liver into the video feed were compared, with a cohort of surgeons.

While much research has been conducted exploring the potential of AR systems for surgery, more work is needed to improve their reliability and usability [9], [26]. One of the key challenges is transitioning from evaluating this research in lab environments in the preliminary stages to evaluating these systems in an ecologically valid clinical environment. Evaluating novel technologies requires a careful balance between engineering and medical requirements [10]. At the same time, it is important to consider the ecological validity of the evaluation. While the operating theatre can be a complex environment and the workflow of these environments should be considered [7], [8], careful attention needs to be paid to the broader impact of interventions. It is also vital to involve clinical professionals in all stages of the project to ensure it meets their needs [27].

### 2.2 Representing surgeons in evaluation studies

It is widely recognised in HCI that studies should test with representative users to ensure we are designing to meet their needs [28]. However, in the context of a hospital, there are constraints which need to be considered [10]. Primarily, it can be difficult to recruit participants with a clinical background, particularly surgeons, as they are usually time-constrained [7], [8]. Such constraints can result in studies with smaller scope and sample size due to the limited availability of surgeons. This can make research in earlier stages challenging, particularly for traditional HCI studies evaluating multiple different prototypes or concepts to explore the design space.

To overcome this challenge, there might still be a place for running preliminary HCI research evaluations with non-representative users [29]. However, for surgeons in particular, there can be a significant gap in performance and cognitive load even between experienced and novice surgeons [30], [31]. Previous work has also indicated differences in gaze patterns between these two groups [32], [33], showing that experienced surgeons had more focused gaze on the key target areas. While that work highlights the importance of ensuring surgeons are included in user studies, it was primarily focused on quantitative performance differences between experts and non-experts. To-date, little work has explored these differences in terms of qualitative feedback.

## 3 Evaluation study

To address the knowledge gap identified from our review of previous studies, **we aim to gain insights on the validity of using engineers for evaluating systems by focusing on understanding the differences between engineers and surgeons in terms of performance, feedback, and preference**.

**Fig. 1:**
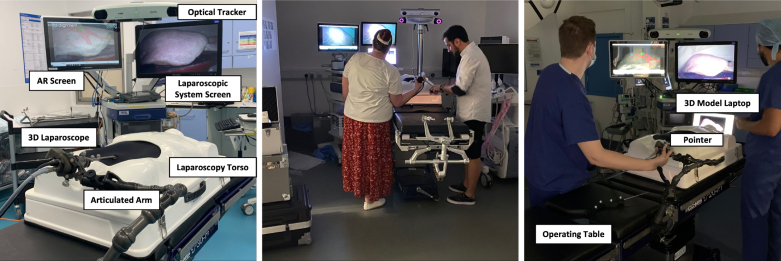
Surgical simulation setup used in this study. Left: The setup used in operating theatres for surgeons. Middle: Example of engineering participant undertaking study in the mock operating room. Right: Example of surgeon participant undertaking study in hospital operating theatre.

The performance and feedback of these two different user groups was evaluated through an experiment aimed at comparing the performance of participants identifying tumour locations within an anatomically realistic silicon liver model between three different AR conditions (Baseline, Split AR and Full AR), as described below. This study was given ethical approval by our local Research Ethics Committee (Z6364106/Computer Science/2022/04/12).

### 3.1 Smartliver AR system

For our study we used a modified version of the Smartliver, which is an existing prototype image-guidance tool which enables AR during laparoscopic liver surgery using an optical tracking system [4], [12]. The hardware setup contained the Smartliver PC (Cybernet medical grade PC), a laparoscopic stack with a Viking 3D laparoscope^2^ and screen, the Smartliver calibration rig, an optical tracking system, an optically tracked pointer for tumour identification, an articulated arm to hold the laparoscope and a laptop displaying the 3D surface of the silicon liver model and tumours. The physical silicon liver model was provided by Health Cuts^3^ and the 3D surface was obtained through thresholds and manual editing using ITKSnap.^4^

In terms of software, the system consists of a Graphical User Interface (GUI) developed on top of Scikit-Surgery libraries [34] with three main widgets: a calibration widget to calibrate the laparoscopic camera to the optical tracking system, an alignment widget to align the 3D surface onto the camera view, and a navigation widget to display the overlay in a 2D image. Even though the system uses a 3D laparoscope, only 2D displays are shown, and the 3D surface is projected onto the Smartliver screen.

### 3.2 Study setup

Fig. 1 left provides an overview of the study setup. The study was conducted between two different locations, the Mock Operating Theatre at our University campus and real but vacant operating theatres at the Royal Free Hospital (London, UK). In both locations we set up a realistic silicon liver model with dimensions 290 × 118 × 126 (mm) that is positioned inside an abdominal laparoscopic torso in an anatomically realistic position. To enable AR in this setup, a tracked instrument was used to localise 4 predefined anatomical landmarks and then align the 3D surface of the phantom to the optical tracker space. This process always resulted in an error below 3 mm. Two different monitors were then set up — the right screen belongs to the laparoscopic system stack and always shows the view of the laparoscopic camera and the left screen shows the three different conditions (Baseline, Split AR, Full AR) which we describe further in the next section. To track the AR view, we had an optical marker attached to the 3D laparoscope [10], that was calibrated using the rig reported in Dowrick et al. [35].

Based on the location, two different laparoscopic system stacks were used as sterilisation restrictions did not allow the same setup to be used in the clinical setting and University laboratory setting. Regardless of these differences, the core functionalities of the AR system were the same.

#### 3.2.1 Mock operating room

Due to restrictions around public access to hospital operating theatres, the engineer user group participated in a mock operating theatre in the university (Fig. 1 Centre). The room we used is a dedicated space for research studies to simulate an operating theatre as closely as possible to maximise ecological validity [10]. This group used a Viking 3D laparoscope and a NDI Polaris Vega (https://www.ndigital.com/) for optical tracking.

#### 3.2.2 Hospital operating theatre

The surgeon group participated in the study in multiple operating theatres used for hepatobiliary surgery (Fig. 1 Right). The use of different vacant operating theatres was due to availability at the hospital. To set up the study in each operating theatre, we needed to move all the hardware to the theatre and set it up (which took 45–60 min each time). Also, due to hygiene requirements, researchers needed to change their clothes and wear scrubs and indoor shoes to prepare the study every time they accessed the theatre.

To work with the availability of the surgeons, who have tight schedules and inconsistent working hours, we needed to set up the study in an off-peak period (between 3 pm–7 pm), when participants have finished their clinic and surgery, and before they attend the night shift. This group used a Storz^5^ 3D laparoscope and a NDI Polaris Spectra^6^ for optical tracking.

**Fig. 2:**
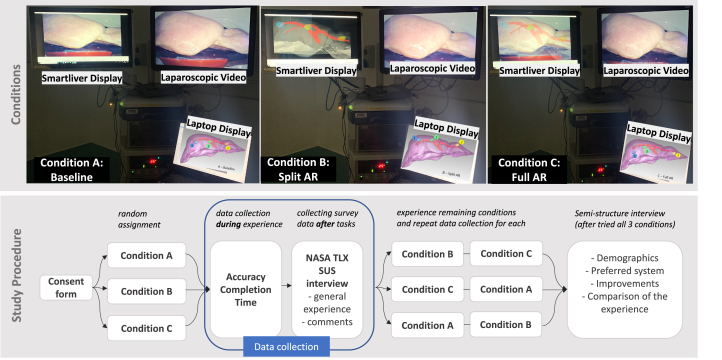
Top: AR conditions considered in our Study setup. For each condition, the Smartliver PC (top left screen) shows a different image guidance visualisation. The locations of the three virtual tumours are also displayed in a grey background. Bottom: Description of Study Procedure. Firstly, participants filled out the consent form. Then, the tumour localisation task was performed under one randomly assigned condition, and quantitative localising accuracy and completion times were extracted. The participant then filled out SUS and NASA TLX forms and was asked to comment on the experience. The procedure was repeated for the remaining conditions and a final semi-structured interview was conducted.

### 3.3 Study tasks

We designed a within-subject user study, with the aim of comparing the performance of participants using the Smartliver AR system to intra-operatively locate the positions of 3 distinct virtual tumours across three different AR conditions (Fig. 2) that differed in the display that is presented to the participant user in the Smartliver screen (left screen in the setup):

(A) “Baseline”: Participants were provided only with the 3D surface information on a laptop, and the Smartliver screen only showed the laparoscopic video feed without the overlay, which is exactly the same as in the laparoscopic screen. This condition aims to emulate the standard surgical approach where the surgeon inspects a pre-operative image of the patient on a laptop and then performs surgery while using the video intra-operatively.
(B) “Split AR”: The Smartliver screen now shows the aligned 3D surface against a black background (i.e., no video).
(C) “Full AR”: The Smartliver screen shows the overlay of the 3D model superimposed on the video picture of the liver. This is the typical AR scenario, where image-guidance is provided by displaying the 3D model aligned with and overlaid on the video.

The order of the conditions was varied across participants to give balanced order of use, to account for potential order effects. For each condition there was no time limit; and while the task given in each condition was the same, the tumour locations were different. The tumour order for each condition followed a colour-coded pattern − the first tumour to find is blue, the second is yellow, and the third is green. The tumour locations were chosen to be superficial so that they could be localised with the tracked pointer, and evenly spread out in the liver to be representative of lesion locations; the lesions were coloured: blue in the right lobe, green closer to the intersection between lobes, and yellow in the left lobe. To ensure that the defined locations were realistic, we obtained feedback from laparoscopic surgeons in our team.

### 3.4 Recruitment

Participants were recruited from our University’s mailing lists, and social networks such as a WhatsApp group within the hospital network to recruit surgeons. When we sent our invitations we also attached a participant information statement for participants to read before making the decision to participate; all participants voluntarily took part in the experiment and initial contact had to be made by them. The order randomisation required multiples of 6 participants. We ended up recruiting 12 participants per group.

**Fig. 3:**
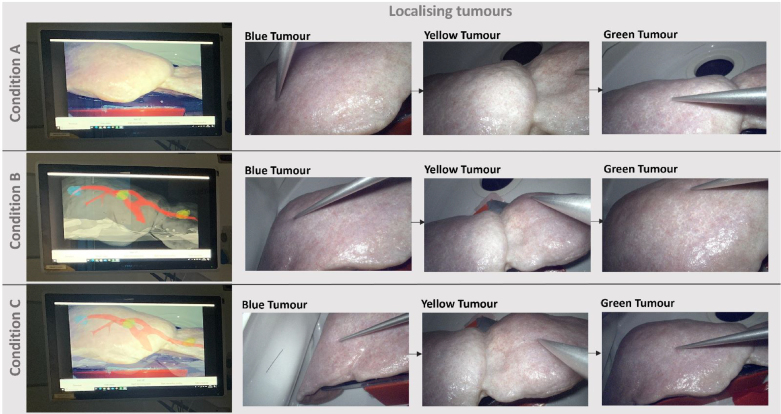
Examples of Tumour localisation under three different conditions (Condition A: Baseline, Condition B: Split AR, and Condition C: Full AR). Smartliver AR system display (from the top left of Fig. 2) per condition is shown on the left, and each row shows the resulting ordered localisation attempt with the pointing instrument at the blue, yellow and green tumours. Note that the displayed view does not correspond to the view that the participant was experiencing at the moment of localising.

### 3.5 Study procedure and data collection

Throughout the experiment we collected both quantitative and qualitative data, following a mixed-methods approach [36]. The overview of the study procedure and data collection are illustrated in Fig. 2.

We first invited each participant to the experiment room and explained to them the overall experiment and their tasks, which were localising three tumours across three different conditions (see Fig. 3). A participant could withdraw at any time without giving a reason, and have any data relating to them removed from the study. If the participant wished to proceed, the researcher then asked for their written consent before beginning the study. The researcher went through a brief tutorial of tools the participant needed to use, such as the laparoscopic camera and instrument for pointing out the tumour locations. We also explained how to hold the tools to ensure the system tracked their movement accurately. Participants were then randomly assigned to one of the three conditions to start with; across all the conditions participants were asked to freely view the liver model 3D surface with tumour locations using the laptop that we provided; then researchers unlocked the laparoscopic camera for participants to find the first tumour location, starting with blue, then yellow and finally green. The order of the colours remained the same. For each condition however, the exact location of these coloured tumours was slightly different (Fig. 2), but in the same anatomical region.

Once a participant was satisfied that they had found the blue tumour location, they let the researcher know, and the researcher then locked the static arms to make the laparoscopic camera steady. With the tracked pointer that we provided, the participant pointed to the location of the tumour; we collected total task time and the accuracy of the identified tumour location. This information was recorded in anonymised form and used to understand performance. Furthermore, the researcher observed the participant’s interactions with the prototype and took note of their behaviours. For each condition, after participants finished with the blue location, they repeated the task for the yellow and green tumour locations. Following the trial of each condition, participants were asked to fill out a System Usability Scale (SUS) [37] to measure the usability of the system, and a NASA Task Load Index (NASA TLX) [38] for measuring their mental workload. After the questionnaires, participants were asked to complete a short interview to provide feedback about their experience.

Following all three tasks, the researchers ran a final interview to collect demographic information including the participant’s experience with laparoscopic liver surgery and familiarity with AR. They were also asked about their overall preferences, suggestions for improvement, and final comments. Interview data was handwritten and audio recorded; this information was transcribed by the researchers following the interview.

### 3.6 Data analysis

To quantify task performance per condition for each participant, we measured the mean localisation error across all tumours in millimetres (mm). For the remaining quantitative data which includes SUS, NASA TLX and logging interactions, we performed statistical tests between the same conditions from different participant groups to test whether the distributions obtained from the engineers were comparable to the ones obtained from the surgeons. Specifically, we performed Wilcoxon signed rank tests to compare distribution medians of performance and SUS scores, and t-tests to compare distribution means of NASA TLX. We chose two separate tests as we found the NASA TLX to follow a Gaussian distribution, unlike the performance and SUS.

To find and meaningfully report patterns within our qualitative data, two members of the research team collaboratively performed an inductive thematic analysis [39] using Miro^7^ a digital whiteboard, to code the audio transcription from the interview questions for each condition and the post study interview.

## 4 Results

We recruited 24 participants across five age brackets, with 16 male and 8 female. Table 1 provides an overview of the participant demographic information. To distinguish between the two participant groups, engineer and surgeon participants are denoted as “<*group name*>*#*” (e.g. engineer #5, surgeon#7, etc.). One surgeon and six engineers had prior AR experience, but this did not affect their results. None of the engineer participants had experience with laparoscopic surgery, but they all conducted research in healthcare-focused engineering. Among the twelve surgeon participants, there were four who had less than three years laparoscopic surgery experience, while the other eight had more than six years experience with laparoscopic surgery, particularly with liver surgery.

**Table 1:** Overview of participant backgrounds for engineers and surgeons group (age group, gender, occupation, AR experience, and year of laparoscopy experience). E = engineers, SS = senior surgeons, and JS = junior surgeons. The top table is the engineer group and the bottom is the surgeon group.

[*Engineers*]	1	2	3	4	5	6	7	8	9	10	11	12
Age group	26–30	26–30	18–25	40+	26–30	26–30	18–25	31–35	40+	31–35	26–30	18-25
Gender	M	M	M	M	F	M	F	M	M	F	F	F
Occupation	E	E	E	E	E	E	E	E	E	E	E	E
AR experience	No	Yes	No	Yes	Yes	No	No	Yes	No	Yes	No	No
Laparoscopy experience	–	–	–	–	–	–	–	–	–	–	–	–

[*Surgeons*]	1	2	3	4	5	6	7	8	9	10	11	12

Age group	31–35	31–35	36–40	26–30	31–35	26–30	36–40	26–30	26–30	26–30	26–30	26-30
Gender	M	M	M	F	M	M	M	M	M	M	F	F
Occupation	SS	SS	SS	JS	SS	JS	SS	SS	SS	SS	JS	JS
AR experience	No	Yes	No	No	No	No	No	No	No	No	No	No
Laparoscopy experience	9	12	11	3	10	3	11	13	10	6	2	1

### 4.1 Usability

**Fig. 4:**
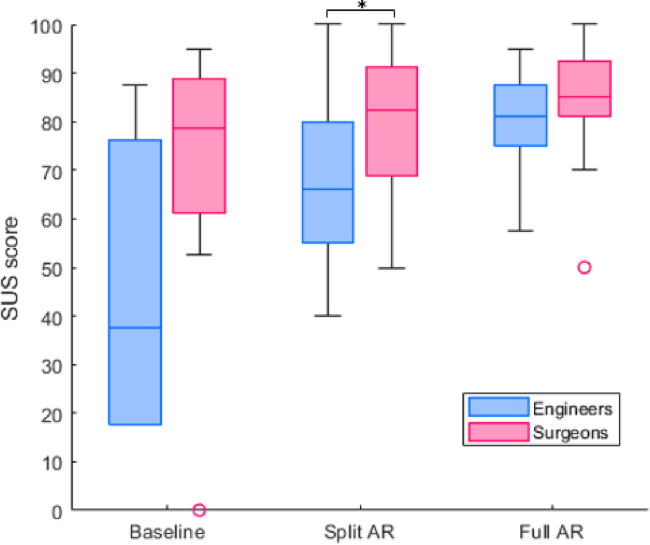
System Usability Scale (SUS) results for the three conditions across the two groups. Black bar with asterisk indicates statistically different distributions with p<0.05 obtained by Wilcoxon test.

According to Lewis and Sauro [40], a mean SUS score of 68 is considered to be average. Fig. 4 shows the results of our descriptive data analysis of the SUS scales across the three prototype representations with two groups. Overall, for the Baseline condition (A) Wilcoxon results show that the two groups have a large but not statistically significant gap (p = 0.055), where the SUS score for engineers was below average (Median = 47.5, SD = 29.4), while the surgeons scored above average (Median = 78.8, SD = 26.3). The results between these groups grew closer in the Split AR condition (B), with engineers providing a slightly below average SUS score of (Median = 66.3, SD = 29.3) and surgeons again scoring well above average (Median = 82.5, SD = 15.4). The gap became even smaller in the Full AR condition, with above average scores for both the engineers (Median = 81.3, SD = 10.3) and surgeons (Median = 85.0, SD = 13.4). For the AR-based options, Wilcoxon tests show that only the Split AR option shows statistically significant differences (B with p = 0.044 and C = 0.47).

### 4.2 User feedback

Interviews with participants revealed both common and divergent themes (reliance, difficulty and preferences). In this section we contrast the two user groups and themes which were identified from a thematic analysis of their feedback across the three conditions.

#### 4.2.1 Reliance

Throughout the feedback we collected from engineers and surgeons across each condition, the word “*confidence*” was mentioned at least once by each participant. Comments from surgeons suggested that their confidence in locating the tumour came from their own experience and ability rather than from the prototype system. For instance in the Baseline condition, eight surgeons mentioned that even though there was no guidance they were still confident with using the prototype as they could relate the position of certain tumours which were close to specific liver surface indentations and edges that were visible in the 3D laptop display. For example, surgeon#12 mentioned that *“I looked at the 3D surface model again and I tried to use landmarks [reference points] to pick where the tumour was, whereas for the other two I just sort of went with it”*. For the Split AR condition surgeons commented that the locations of the tumours were difficult to identify, giving them less confidence in the system. According to surgeon#8, *“I think just because where the lesion is positioned, there’s just a lot of liver around that area [...] like with the laparoscope, you’re only seeing the small, you’re not seeing the whole of it, only part of it [only a small region of the liver can be seen].”.* In the Full AR condition, participants tended to rely on the AR overlay, and the comments were directed towards the task itself. From surgeon#9 *“Because the image is superimposed on that liver I think you can’t go wrong”*. Therefore it seems that without AR surgeons rely on their experience, and once AR is introduced they rely on the system.

On the other hand, engineers commented that their lack of confidence was due to their perception. The engineers completed the tasks for the Baseline condition in the same way as the surgeons, i.e. trying to find reference edges and indentations to determine the tumour location. However five of the engineers could not locate any reference points. For example, engineer#6 mentioned that *“there was no identifiable mark on the liver which made it very difficult for me to find out specifically, it’s more of a guess”* and engineer#11, *“[...] you could see where the tumour was you have to get up into like the valley [indentation in the liver] for the green tumour, and so on. So that helped a lot in enriching the correct location of the tumour”*. The comments themselves were quite similar to those of the surgeons in the Baseline, the main difference being that the engineers lacked ’perceived’ confidence. In the Split AR and Full AR conditions, four engineers mentioned that they mainly relied on and trusted the system to complete their tasks. For instance, engineer#7: *“I felt like I was trusting the augmented model”*. This trust remained with that participant despite the AR overlay not always being perfectly aligned, *“[...] even if I didn’t feel it was properly superimposed, I felt it was much easier to identify everything”*.

The other engineers were critical of the system and the content’s alignment. For instance, in Condition B engineer#2 *“I don’t think it was well aligned. If I have to be honest, the registration wasn’t good”*. These issues persisted in Condition C also with engineer#5 commenting *“the shape wasn’t matching properly. And there was a misalignment in the overlays as well. Which might be difficult to know”*. Lag was also a factor in this condition according to engineer#6 *“because the overlay had some kind of like lag, it was hurting my hands to look at it constantly. So I would look at the other one. And I would position the needle where I wanted it to be when I was looking at the overlay”.*

However, surgeons seemed to be more forgiving about this, with no specific comments related to alignment. This suggests that surgeons are more willing to overlook such system issues due to their lower reliance on the system instructions.

#### 4.2.2 Difficulty

Six engineers and one surgeon commented that for the Baseline prototype it was quite difficult as it was too abstract to interpret without a guide. Engineer #10 *“I felt I was close to it, but I just found it a little bit hard to pinpoint [the tumour] using the stick [pointing instrument] while referencing the image [the 3D model of the liver on the laptop display] essentially”*, while surgeon#10 mentioned that *“without AR it was difficult to mark the surface, even though I have a rough idea about the segment. It’s always difficult without tactile sensation”*.

In the Split AR condition, participants started feeling that the tasks were easier due to the reliability of the system and they could get help while completing the tasks. Five participants (4 engineers and 1 surgeon) commented that it was easy. Engineer #7 mentioned that *“it’s easy to locate everything. You’re seeing the same thing. Even if the needle (pointer) wasn’t there. You were seeing the same thing. So it was easier to point out what I thought was”*. While a surgeon participant felt it was easy and helped as they did not need to see too many screens (surgeon#6 - *“it was easier because I saw something in the surface of the liver and don’t need to see the laptop*”).

While participants still commented on the difficulty experienced by split attention between the screen and their dummy patient, an engineer participant (engineer#12) mentioned that “*I found it really difficult. And I found that I had to kind of understand the visuals and like the pattern of the liver on the left screen and then map it on to the right*”, and the AR overlay made it easier to do the task, as surgeon#6 noted: “*it was easier because I saw something in the surface of the liver and don’t need to see laptop”*.

Seven participants commented that with Full AR they felt the tasks were easy as they could see the liver image and live feedback while they were doing tasks, and no participants mentioned that it was difficult. As pointed out by surgeon#10 *“[...] the image and the real negative is superimposed over the livers. It is really helpful. It is really easy to pinpoint”*.

In terms of how well the system worked, for both AR prototypes the main comments were on the alignment and overlay which were only noticed by six of the engineers. For instance, engineer#2 mentioned that *“I kind of need to find a way to compensate for the errors in the lining [alignment]. Otherwise, it’s just misleading”*. However participants also commented that the AR system could make the task less complex; for instance, surgeon#12 mentioned that, “*I think having the overlap of the virtual image with the tumours was really helpful and you can find the landmarks, I know the same thing on that screen before, but it’s helpful to see it overlapped on to the actual liver. It was easier to landmark*”. Furthermore, AR seemed to reduce the complex split-attention nature of the setup, surgeon#3 “*if you’re viewing in two dimensions [referring to Condition A], then you can’t appreciate whether it’s anterior or posterior, but seeing the augmented reality on the laptop, you could navigate and see whether it’s on top, anterior or posterior termination to the blood vessel*”.

#### 4.2.3 Preferences

The analysis of the data from the interviews shows that all of our participants from both groups would like to adapt and use it for the future operating theatre. None of the participants preferred to have Baseline (A) setting, despite our expectation that some surgeon participants would prefer this. Overall, the most popular system was Full AR with 15 participants preferring this (6 engineers and 9 surgeons). There were 7 participants who preferred a Split AR prototype (5 engineers and 2 surgeons) and 2 participants who preferred both Split AR and Full AR prototypes (1 engineer and 1 surgeon).

• **Split AR** - The seven participants who preferred this condition mentioned it was due to it being (1) “helpful”; (2) “less busy”; and (3) in a “more realistic setting”. Three engineer participants specifically mentioned that Split AR was the most helpful setting as the screen was less busy and the AR guidance is there for just the second screen. Another factor that participants liked about this prototype was its realistic setting, meaning that it is set up to closely resemble a real operating theatre. For instance, engineer#11 mentioned that *“I think it gives you a better mix of what the surgeon are used to”*. Surgeon#5 expanded on this perspective, *“I think it just helps a lot with localising tumours. So if you’re able to map it with a patient’s CT scan that you’re going to operate on, seeing exactly where you’re going to be doing the resection would be hugely beneficial”*.
• **Full AR** - Most of our participants preferred to use the Full AR condition (6 engineers and 9 surgeons). Three engineer participants commented that they prefer this condition as it was very easy to use. All other participants mentioned that they prefer this system as it supports visual feedback during the tasks. Engineer #6 reported that *“It gave me everything I needed. I only needed it on one screen [...] with the overlay, it kind of gives me a different sense of perception of where some parts of the liver actually are. The overlay also gives me a better idea of specifically where the tumours are”.* Engineer #12 also commented that *“because it gives very good feedback on where the tumour was and I could see the instrument both in the actual appearance of the camera and in the augmented reality overlay”.* The visual support from the AR overlay helped participants to identify the location of the tumours. Surgeon#2 mentioned that *“the tumour is marked on the liver and it’s exactly an imprint of the liver that I see. So obviously, we can see we present the anterior posterior segment. So that makes it easy for us to identify”.*
• **Both Conditions (B & C)** - In addition, 2 participants (1 engineer and 1 surgeon) liked both B and C systems. Two engineer participants mentioned that they felt more confident using split AR but Full AR makes it much easier to identity things faster - engineer#7, “*I felt more confident with the split AR but I realised that the full AR one was the easiest one, the fastest one to identify things*”. One surgeon participant also commented that both systems are similar and potentially very important in the laparoscopy, particularly mentioning that it could be really helpful - surgeon#6, *“I think that the second (B) and the third (C) system, it was similar. The only difference was that I need to stop and look again, in the third part. The third task, I needed to stop and look again to look up but it wasn’t so demanding to do that”.*

### 4.3 Tumour localisation performance

**Fig. 5:**
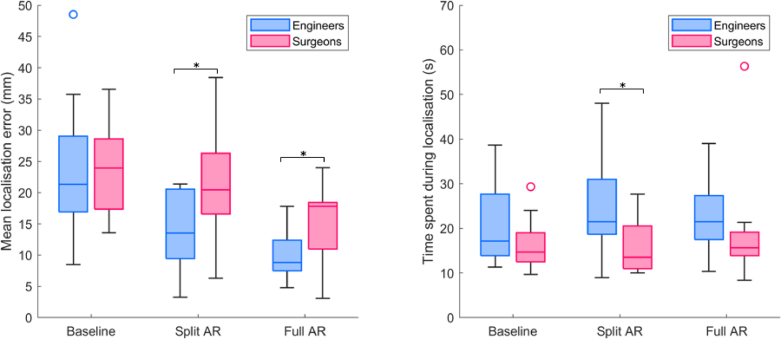
Left: Mean localisation error across 3 tumours for three conditions (A-Baseline; B-Split AR; and C-Full AR) and two groups of participants. Right: Time taken for tumour localisation for three conditions and two groups of participants. Black bar with asterisk indicates statistically different distributions with p<0.05 obtained by Wilcoxon test.

The tumour localisation performance for each participant group, along with the mean localisation accuracy and task time expense per condition, are presented as boxplots in the left and right side of Fig. 5, respectively. For the baseline condition A, Wilcoxon test results show that the localisation error for engineers (Median = 23.9, SD = 7.1) and surgeons (Median = 21.3, SD = 10.7) are not statistically different with p = 1.000. For the AR conditions (B & C), localisation error shows statistically significant lower values (B with p = 0.012 and C with p = 0.043) for engineers (B with Median = 13.5, SD = 6.1 and C with Median = 8.8, SD = 4.0) than for surgeons (B with Median = 20.4, SD = 8.6 and C with Median = 17.8, SD = 6.3). Across conditions, for both groups the localisation accuracy Median values decrease in the same trend of A < B < C, meaning that the lowest error and therefore highest accuracy were achieved for the full AR condition. For localisation time expense, Wilcoxon only found significant differences between groups for condition B, where surgeons spent less time (Median = 13.5, SD = 6.3) than engineers (Median = 21.5, SD = 11.1). For the remaining conditions, the same difference is observed across groups but without statistical significance, with condition A showing from (Median = 17.2, SD = 9.6) to (Median = 14.7, SD = 5.8) and condition C from (Median = 21.5, SD = 8.1) to (Median = 15.7, SD = 12.3). These results indicate that surgeons spend less time than engineers to complete the tumour localising task.

**Fig. 6:**
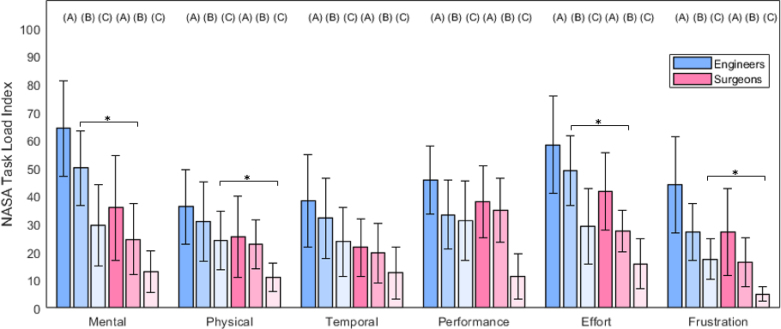
NASA TLX for 3 conditions (A-Baseline, B-Split AR, C-Full AR) from two groups of 12 participants. Each bar height refers to the mean value across 12 participants and the error bars to half the standard deviation. Black bar with asterisk indicates statistically different distributions with p<0.05 obtained by t-test.

NASA TLX values obtained from questionnaires from both groups are presented in Fig. 6. For each of the six criteria, we present a bar plot with mean and standard deviation (scaled to half for visualisation purposes) for each condition and each group of participants. Overall, across similar conditions, mean values are smaller for surgeons than for engineers. The only exception to this trend is the perceived Performance in condition B, where engineers obtained M = 33.0, SD = 24.7 and surgeons M = 35.0, SD = 23.1. Compared to the quantitative localisation error results, both groups show decreasing values across conditions A, B, C, showing that workload and surgical task performance are in agreement.

To better understand whether there are significant differences between the groups in terms of workload, we present the p-values Wilcoxon tests between surgeons and engineers within fixed conditions in Table 2. None of the workload values show statistical difference for the Baseline condition A. For condition B, Mental demand and Effort values are significantly smaller with surgeons (M = 24.5, SD = 25.3, and M = 27.5, SD = 14.8) than engineers (M = 50.0, SD = 26.4, and M = 49.2, SD = 24.9). The same effect is observed for the perceived Performance and experienced Frustration in condition C, but with smaller absolute differences — engineers (M = 31.2, SD = 28.1 and M = 17.5, SD = 14.4) and surgeons (M = 11.3, SD = 16.0 and M = 5.0, SD = 5.2).

**Table 2:** List of t-test p-values comparing NASA TLX values from engineers to surgeons with fixed conditions. Row indicates the condition being compared, columns the NASA TLX criterion. Bold values indicate statistical significance (p<0.05).

	Mental	Physical	Temporal	Performance	Effort	Frustration
A - Baseline	0.1161	0.425	0.142	0.435	0.210	0.210
B - Split AR	**0.0266**	0.462	0.268	0.765	**0.030**	0.136
C - Full AR	0.124	**0.035**	0.141	0.060	0.155	**0.023**

## 5 Discussion

Our work aimed to understand whether engineers could be a viable alternative to surgeons as participants for preliminary studies of surgical systems. Based on the results of this study we gained insights into the differences between these groups, specifically around the difference in system expectations, the risk of over- and under-performing, and how increased reliance on the system can mean greater performance similarities.

### 5.1 Difference in system expectations

Engineers were more critical of imperfections when it came to registration accuracy. This could be due to their training (as engineers) as well as their stronger reliance on the AR system compared to surgeons. The surgeon group were less critical of the system, seeming to be less reliant on perfect AR overlay alignment — probably due to surgeons drawing on their experience. This highlights that surgeons saw the AR overlay as more supplementary, and therefore data collected from engineers acting in place of surgeon participants may cause designers to “over-do it” in terms of the system accuracy. This is not necessarily a negative, but there could be a point where engineers are over investing the time in making a system more accurate when surgeons care more about other aspects of the design — therefore it is important to later involve experienced surgeons to ensure the system meets their specific needs [28].

### 5.2 The risk of over- and under-performing

A key difference in the performance between the two groups in our study is that our surgeon participants spent less time than engineers in the localising task — which is partly in line with work by Law et al. [33] which showed that Surgeons are faster and more accurate than novices. Even though surgeons were faster, localisation error performance was similar to the engineers in Condition A and worse for Conditions B and C. Despite this being a positive for the effectiveness of the tested AR system, it suggests that the engineer group is less representative as they were too accurate in localising the tumours, which could reduce the usefulness of their performance data.

This seems to be directly related to surgeons being more experienced in laparoscopic surgery and more comfortable in handling laparoscopic tools inside the abdominal cavity — surgeons positioned the laparoscopic tools much more intuitively than engineers and reached a decision on picking location much faster. They also tended to rely more on their understanding of the pre-operative information (displayed on the laptop) and its relation to the real anatomy of the liver, restricting the level of desired precision to the anatomical liver segments and not the exact tumour locations [41]. This contrasts with the engineers who relied primarily on the AR displays due to less experience with the anatomy and confidence with the laparoscopic setup. Such a difference in confidence is reflected in the time taken to decide on the location.

Another interpretation is that the observed accuracy measurements are not representative of the real surgery case scenario. It could be that our surgeon participants, due to being time-constrained, were mostly focusing on finishing the study as quickly as possible and therefore rushed through their task, or that the provided task information encouraged rapid completion rather than a calculated and slow approach to maximise accuracy. This could indicate that the data from surgeons may not always be reliable and HCI researchers should consider external factors such as time constraints when recruiting surgeons for studies.

### 5.3 Increased reliance on the system can mean increased performance similarities

In addition to the tumour localisation performance measurements and feedback comparison between engineers and surgeons in fixed conditions, we observe that, across conditions, results highlight the same trend — both groups show increasing localisation accuracy and usability in Baseline→ Split AR→ Full AR. This trend is observed with increasing values in SUS and decreasing values in the mean localisation error.

**Fig. 7:**
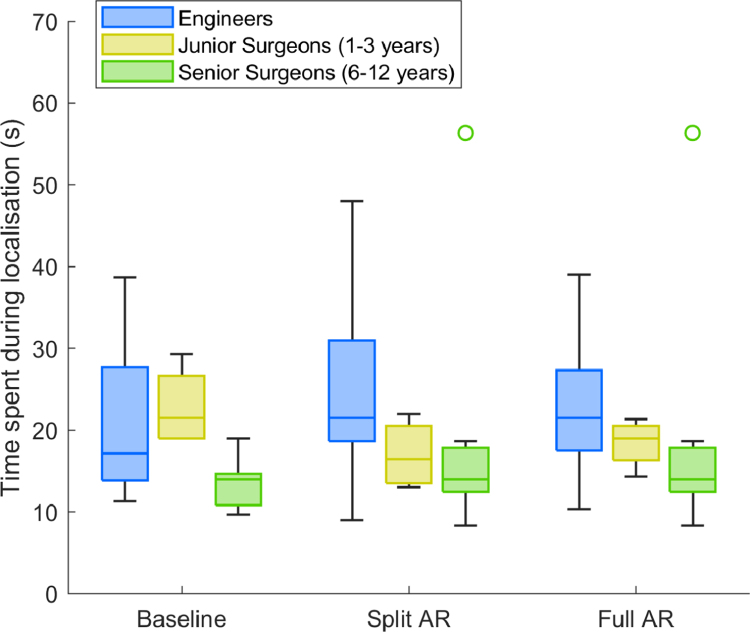
Time taken for tumour localisation for three conditions and three groups of participants (Engineers and two surgeon sub-groups).

Therefore, it seems that surgeons and engineers can build up a reliance on AR information. The engineers had no surgery experience but the AR system could help them perform at a comparable level to the surgeon group. The surgeons used the AR system alongside their existing experience. However, both groups utilised the same localising strategy of using landmarks to identify tumour locations, which is a documented technique that particularly benefits less experienced surgeons [42]. To further understand the effect of surgical experience on reliance, we separated the task performance and time measurements of Fig. 5 of the surgeon group into two sub-groups, Junior surgeons with up to 3 years of experience (4/12) and Senior Surgeons with at least 6 years of experience (8/12). Fig. 7 displays time expense results for the new set of 3 groups (Engineers also included) - in this case, median time results show that junior surgeons are faster than engineers in the task but slower than the most experienced surgeons. This indicates that greater surgical experience can lead to a decrease in task completion time and, between surgeon groups, the less experienced may perform more similarly to the engineers. We omit the obtained localisation error results for the three groups as they did not show any noticeable difference from the 2 group analysis. This indication is potentially relevant for future studies, as AR technologies have potential for providing image guidance mostly to less experienced surgeons by speeding up training and improving performance. Regardless, surgeons may also have other visual strategies based on their experience [32] and these specific differences should be explored further in future research as well.

In terms of preference, conditions B and C were always chosen over A by both groups. When asked about this, participants from both groups mentioned that it was because of the visual feedback and ease of use; this is despite surgeons having relevant expert knowledge and experience. Specifically, in the case of the surgeons, the majority (9 out of 12) preferred Condition C, with two preferring Condition B and one ranking them both equally. In the case of the engineers it appeared that they were split across Conditions B (5 out of 12) and C (6 out of 12), with one ranking them both equally. The AR overlay reduced the perceived workload of participants in both groups according to their responses to the NASA TLX questionnaire.

### 5.4 Reflections

#### 5.4.1 Recruiting surgeons

Along with our findings into the use of data from engineers, we also gained insights on recruiting and obtaining data from surgeons. While recruiting engineers (engineers) was relatively straightforward as there were no specific requirements, recruitment of the surgeons for this study was difficult as we needed to access surgeons who focus on liver surgery. In the hospital we were working with, there were only 18 liver surgeons. Therefore it became challenging to schedule a time with the surgeons as their diaries were full due to heavy demand. Surgeons can often need to drop out of the study due to sudden changes to their schedule and their preceding schedule may run over-time or finish early. Therefore it is important to build flexibility into the study design to account for this. Previous studies have also recognised this challenge [43], [44].

#### 5.4.2 Access to operating theatres and study setup

This schedule uncertainty issue was further exacerbated by the fact that access to the operating theatres was limited. For our study to be ecologically valid [10] with surgeons, the setup needed to be tested in real operating theatres where laparoscopic liver surgery is performed. Access to these rooms was only granted on specific days and times, mainly in the late evening when procedures were finished and premises clean. Therefore, an essential aspect of this study was the engagement with the theatre coordination team: only with close collaboration was it possible to secure operating theatre space, even if the theatres available were different for each day. Theatre availability was not always guaranteed and the same room was not always available. This also implies that the study setup should be flexible enough to be deployed quickly even if room availability is variable in terms of location and time. However a caveat with running studies so late is that surgeon participants could potentially experience fatigue due to their previous surgical work and having limited time availability in their schedule. In this study, 45 to 60 min were required to move the laparoscopic stack from the hospital research facility to the operating room and to set up the software Smartliver AR system and hardware tools (laparoscopic and tracking system), and wear proper clothing (i.e. scrubs). Extra time was necessary to turn off, clean, and move the system back to the research facility. Besides time, this also requires physical workload from the personnel running the study.

### 5.5 Limitations and future directions

This study has some limitations. Firstly, as mentioned in 3.2 Study Setup, the laparoscopic system hardware used by each group of participants was different due to the location requirements of the study and access limitation of the engineers entering the hospital. However the hardware devices are similar and the core functionalities of the AR systems are the same.

It is noteworthy that the availability of surgeons can be an obstacle, as the number of surgeons is limited in each hospital. Working across hospitals can also be challenging as each hospital might have different access conditions. This is a major challenge for HCI studies which focus on clinical contexts [10].

The task being performed is specific to laparoscopic surgery and the results may not be transferable to other types of tasks. Further research is needed to explore this.

Additionally, designing with novice surgeons or engineers could be relevant for future digital surgical interfaces designed for training novices or for supporting collaboration with remote expert surgeons who monitor the procedure and provide feedback in real time [45], [46].

## 6 Conclusion

We have performed the first usability study on the use of AR for laparoscopic surgery during a simulated surgical task testing with both a group of trained surgeons and a group of participants without a clinical background (engineers). Our aim was to understand whether engineers could be participants in place of surgeons in preliminary studies. Our results suggest that engineers and surgeons have some similarities when using the AR system, indicating that engineers could be considered to at least test technologies in preliminary stages to provide insights on performance and preferences. This is significant given the challenges with recruiting surgeons for studies, and could also decrease the percentage of surgeons necessary to test system usability, reduce logistic challenges in recruitment, and ease clinical translation. However, this study has also highlighted important differences in both performance and perceptions between the two groups that should be taken into account in future studies with surrogate users.

## CRediT authorship contribution statement

**Soojeong Yoo:** Conceptualization, Formal analysis, Investigation, Project administration, Writing – original draft, Writing – review & editing. **João Ramalhinho:** Formal analysis, Investigation, Project administration, Writing – original draft, Writing – review & editing. **Thomas Dowrick:** Methodology. **Murali Somasundaram:** Resources. **Kurinchi Gurusamy:** Resources. **Brian Davidson:** Supervision. **Matthew J. Clarkson:** Supervision. **Ann Blandford:** Supervision, Writing – review & editing.

## Declaration of competing interest

The authors declare that they have no known competing financial interests or personal relationships that could have appeared to influence the work reported in this paper.

## Data Availability

Data will be made available on request.

## Footnotes

1 Microsoft HoloLens −https://www.microsoft.com/hololens.

2 Viking 3D laparoscope - www.conmed.com.

3 Health Cuts - www.healthcuts.com.

4 ITKSnap - http://www.itksnap.org/.

5 Storz - https://www.karlstorz.com/.

6 NDI Polaris Spectra - https://www.ndigital.com/.

7 Miro - https://miro.com.
